# Enhanced Adsorption of Metformin Using Cu and ZnO Nanoparticles Anchored on Carboxylated Graphene Oxide

**DOI:** 10.3390/polym18010071

**Published:** 2025-12-26

**Authors:** Abeer H. Aljadaani, Amr A. Yakout, Hany Abdel-Aal

**Affiliations:** 1 Department of Chemistry, College of Science, University of Jeddah, Jeddah 21589, Saudi Arabia; 2 Chemistry Department, Faculty of Science, Alexandria University, Alexandria 21636, Egypt

**Keywords:** copper nanoparticles, metformin, adsorption, pharmaceuticals, carboxylated graphene oxide, reusability

## Abstract

Pharmaceutical residues are increasingly emerging in global drinking water sources, posing serious ecological and public health challenges by altering the physicochemical balance of aquatic systems. Among available purification approaches, adsorption remains one of the most promising techniques due to its simplicity, cost-effectiveness, and efficiency. In this work, a ternary nanocomposite of Cu- and ZnO-decorated carboxylated graphene oxide (Cu/ZnO@CGO) was synthesized and utilized for highly efficient and ultrafast removal of the antidiabetic drug metformin from aqueous environments. The adsorption mechanism arises from a synergistic combination of surface complexation on Cu nanoparticles, cation–π and π–π electron donor–acceptor interactions with the CGO aromatic structure, and hydrogen bonding through the amino groups of metformin and the oxygen-rich functional moieties of ZnO and CGO. The nanocomposite was thoroughly characterized using FTIR, XPS, XRD, SEM, HRTEM, and TGA analyses, confirming its well-defined hybrid structure. Unlike conventional single-phase or binary systems, the Cu/ZnO@CGO nanocomposite demonstrated remarkable cooperative effects that enhanced its performance through the integration of metal–ligand coordination, π–π stacking, cation–π forces, and hydrogen bonding. These interactions contributed to an outstanding adsorption capacity of 232.56 mg·g^−1^ and an exceptionally fast equilibrium time of only 25 min. Moreover, the material maintained excellent reusability, with merely a 4.1% decline in efficiency after five regeneration cycles, and achieved almost complete removal of metformin (99.7 ± 3.4%) from several real water samples, namely river, tap, and bottled water. The unique structural design of Cu/ZnO@CGO prevents CGO aggregation and facilitates efficient contaminant capture even at trace concentrations, establishing it as a highly competitive and sustainable adsorbent for pharmaceutical wastewater treatment. Overall, this study highlights a novel and rationally engineered nanocomposite whose synergistic surface chemistry bridges adsorption and detoxification, providing valuable insight into the next generation of multifunctional graphene-based materials for environmental remediation.

## 1. Introduction

**Figure 1:**
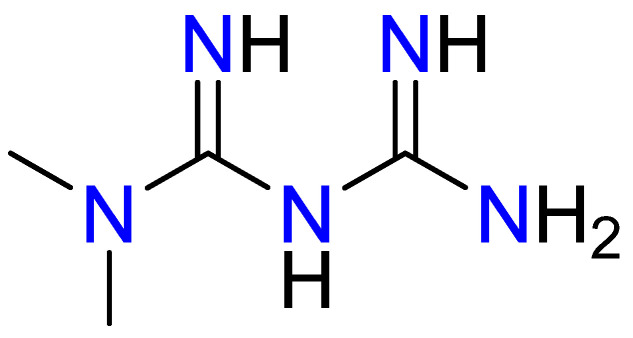
Metformin chemical structure.

The harmful impacts of pharmaceuticals on the environment have drawn considerable attention to their presence in wastewater and groundwater treatment facilities [1,2]. One of the main environmental issues is believed to be the increasing consumption of medications and their improper disposal into ecosystems. Different functional groups found in pharmaceuticals are used to cure and prevent diseases. Following administration, a significant portion of medications are eliminated in an unmetabolized form into wastewater treatment facilities and household sewage. Therefore, the main route that medicines enter water bodies through is the excretion of metabolites and parent chemicals, followed by wastewater treatment. The accumulation of traces of pharmaceuticals in water bodies additionally occurs through surface runoff, sewer leaks, and the disposal of unwanted or expired medications [3,4,5]. As a result, these types of pollutants must be removed from the aquatic environment. One of the efficient medications often used globally to treat type 2 diabetes (non-insulin dependent) is metformin, also known as 1,1-dimethylbiguanide [6]. Since over 150 million individuals take metformin each year, it is one of the main medications found in sewage effluent [7]. Polycystic ovarian syndrome, an endocrine condition, can also be successfully controlled with metformin [8]. Approximately 30% of metformin is digested within the body, with the remaining 70% being excreted in urine [9]. The metformin concentration in sludge treatment plant wastewater influents has been measured in the Netherlands and found to be 73.73–84.41 μg.L^−1^, respectively [10]. Moreover, researchers found guanylurea, a byproduct of metformin’s biodegradation, in surface water and STP effluents, with concentrations ranging from 56–39 and 3.9–1.8 μg.L^−1^, respectively [10]. Metformin has also been detected in the wastewater treatment plants’ influents in the USA and found to be in the concentration range of 3.2–100 μg.L^−1^ [11]. In the influents of German treatment plants for wastewater, metformin and guanylurea were also found to be present in concentrations between 18 and 105 μg. L^−1^ and less than 0.25 and 3.0 μg. L^−1^, respectively [12]. In Australian and Malaysian wastewater treatment plants, metformin has been detected in the effluents at a concentration level of 2–53 μg. L^−1^ [13] and 34.228 μg.L^−1^ [14], respectively. Additionally, Portugal’s wastewater treatment plants have found higher levels of metformin. (70–325 μg.L^−1^) in their effluents [15]. Once metformin is continuously detected in wastewater and absorbed into streams and rivers, it may accumulate in people and result in vitamin B12 deficiencies or lactic acidosis [16]. Additionally, it has been observed that adult male fish exposed to environmentally relevant amounts of metformin exhibit endocrine disruption [17]. However, no agency has yet established the lowest acceptable limit for drinking water. As a result, metformin has become an environmental issue, necessitating the development of easy-to-use methods for removing metformin residue from water. Pharmaceuticals were extracted from aqueous systems using a variety of water treatment techniques, including coagulation [18], biological treatment [19], photocatalytic degradation [20], catalytic ozonation [21], electrochemical advanced oxidation [22], adsorption, and more [23,24,25]. Due to its ease of use and extensive choice of adsorbents, adsorption is regarded as one of the effective methods with the greatest promise for removing various contaminants [26]. A variety of adsorbents have been used to remove metformin from water, including activated carbon [27,28], magnetic carbon composites [29], iron-modified seed husks [30], silica-alumina composite [31], biochar [32,33], and natural clay [34]. To remove contaminants from aquatic sources, graphene oxide (GO) has also been used as an excellent 2D-carbonaceous adsorbent [35,36] and is mostly produced via oxidative exfoliation from graphite [37]. Many oxygen-containing groups, including carboxyl, hydroxyl, and epoxy groups, are present in the highly oxidized graphene layer. Because of these distinctive functional groups, GO is an extremely effective adsorbent in the field of pollution cleanup. GO has also been widely used as a super adsorbent due to its remarkable physical and chemical properties, layered structure with huge theoretical specific surface area, strong π-electron mobility, and affordability [38]. Although GO has been used to remove metformin from water-based solutions, its removal efficacy is limited due to aggregation in aqueous environments [39,40]. This occurs because of strong π–π interlayer characteristics and van der Waals interactions between the surrounding sheets, which reduce surface area and limit contaminant uptake [41,42,43,44,45]. High-speed centrifugation is necessary to extract GO from water because of its low specific weight and fine particle size [46]. Other substances have been added to GO to produce nanocomposite materials to remove these drawbacks and improve the adsorption behavior [47]. Several investigations have focused on carboxylating graphene oxide (CGO), which has a higher carboxyl group concentration and a wider interface distance, to improve the adsorption capability of GO [48,49,50]. One of our ongoing research projects is exploring new nanosorbents for water reuse that utilize functionalized *G* or GO NPs to filter out various water pollutants. GO and its derivatives have been extensively explored as nanosorbents for pharmaceutical contaminants; however, conventional systems often face drawbacks such as particle aggregation, restricted adsorption capacity, and sluggish kinetics. Earlier investigations largely focused on either Cu- or ZnO-modified graphene oxide individually, yet no attempt has been reported so far on the simultaneous incorporation of both Cu and ZnO nanoparticles within a CGO framework for metformin removal. This ternary nanohybrid is designed to address CGO’s tendency to aggregate, while offering diverse and complementary adsorption pathways, including Cu-mediated complexation, ZnO surface binding, and π–π/EDA interactions facilitated by CGO. Importantly, this study introduces, for the first time, a Cu/ZnO@CGO composite that achieves rapid and highly effective adsorptive removal of metformin (Figure 1) from aqueous systems. Herein, the anchored Cu- and ZnO-NPs in the CGO can interact with the electron-donor nitrogen atoms (amino, imine, and dimethylamine groups) in metformin to produce stable metal–metformin complexes, which greatly improve metformin removal efficiency. The mechanisms governing the novel nanocomposite’s rapid and effective metformin adsorption were deduced from the impacts of pH, ionic strength, kinetic models, and fitting sorption isotherms. Moreover, metformin was successfully removed from various environmental water samples treated with the Cu/ZnO@CGO nanocomposite.

## 2. Experimental Section

### 2.1. Materials and Reagents

Zinc acetate dihydrate (Zn(CH_3_COO)_2_·2H_2_O), copper nitrate trihydrate (Cu(NO_3_)_2_·3H_2_O), L-ascorbic acid, NaCl, and Chloroacetic acid were analytical reagent grade (Sigma-Aldrich, St. Louis, MO, USA). Analytical-grade metformin hydrochloride (99.99%) was acquired from Selleck Biotechnology GmbH. The Chemical Reagent company of Sinopharm provided urea (H_2_NCONH_2_) and graphene oxide powder. A 500 mg L^−1^ metformin stock solution was obtained by dissolving the calculated amount of the compound and preserved at 4 °C. Each day, the required concentrations of working solutions were freshly prepared by diluting the stock solutions with Milli-Q water.

### 2.2. Instrumentation

FTIR spectra were recorded using a Nicolet 400 Fourier transform infrared spectrometer. UV–Vis analyses were conducted with a Labomed Inc. spectrophotometer employing a 1 cm quartz cuvette. X-ray diffraction patterns were obtained using a powder X-ray diffractometer equipped with a Cu Kα radiation source (λ = 1.5418 Å; D/MAX-2550, Rigaku, Tokyo, Japan). The morphology of CGO and Cu/ZnO@CGO nanocomposites was examined using high-resolution transmission electron microscopy (HRTEM, JEOL JEM-2100V) and scanning electron microscopy (JEOL JSM-6010LV, Tokyo, Japan). To determine the Brunauer–Emmett–Teller surface area and pore size distribution, and to record the nitrogen (N_2_) adsorption–desorption isotherms, analyses were carried out using a Physisorption Nova 3200-N instrument, Quantachrome Instruments, Boynton Beach, FL, USA.

### 2.3. Synthesis of Cu/ZnO@CGO Nanocomposite

The carboxylation of GO, hydrothermal production of Cu-NPs, and ball-mill blending of Cu-NPs, ZnO NPs and CGO comprised the three straightforward stages used for generating the Cu/ZnO@CGO nanocomposite. Initially, 5.0 g of graphene oxide (GO) was treated with 30 g of NaOH and 25 g of chloroacetic acid to introduce carboxyl functionalities by modifying the epoxy and hydroxyl groups located on both the basal plane and edges of GO. The resulting suspension was subjected to ultrasonic agitation for 5 h. The formed carboxylated graphene oxide (CGO) was collected by filtration, thoroughly washed with methanol and deionized water, and subsequently vacuum-dried at 70 °C.

In the subsequent step, L-ascorbic acid was used as an antioxidant, polyvinylpyrrolidone (PVP) served as a stabilizing and reducing agent, and a 0.1 M Cu(NO_3_)_2_ solution was employed as the copper precursor. The solution pH was adjusted to 12, and 0.2 M NaOH was added as a catalyst. The reaction mixture was carefully transferred into a 100 mL Teflon-lined stainless-steel autoclave and heated at 190 °C for 5 h. After completion, the resulting brown-black copper nanoparticle solid was separated by centrifugation, washed repeatedly with ethanol and water, and finally dried in an oven. In a similar procedure, zinc oxide nanoparticles were synthesized hydrothermally by mixing 0.5 mmol of zinc acetate and 2.0 g of urea as starting materials and heated at 180 °C for 16 h. The mixture was then cooled to room temperature and washed several times with distilled water, ethanol and then the white product was calcined at 200 °C for 2 h [51]. Lastly, a stainless-steel ball mill was used to finely blend the Cu, ZnO nanoparticles, and the black CGO nanosheets at a frequency of 25 Hz for 30 min.

### 2.4. Batch Studies to Evaluate Metformin Removal by Cu/ZnO@CGO

In batch experiments, 25 mg of Cu/ZnO@CGO nanoparticles were placed in centrifuge tubes containing a 20 mg L^−1^ metformin solution. To minimize photodegradation of metformin, the suspensions were wrapped in aluminum foil and ultrasonicated for 2 min, after which the pH was adjusted to 6.0 using 0.05 M HCl or NaOH. The mixtures were then agitated at 250 rpm in a temperature-controlled shaker. After separation, the residual metformin concentration in the clear supernatant was quantified spectrophotometrically using a calibration curve constructed from standard metformin solutions, measured at a maximum absorption wavelength (λ_max_) of 235 nm [52].

To evaluate the influence of pH, the suspension pH was varied over the range of 2–11. For adsorption kinetics and removal efficiency studies, 25.0 mg of Cu/ZnO@CGO nanoparticles were added to 10.0 mL of metformin solutions with initial concentrations of 20.0 and 50.0 mg L^−1^. Samples were collected at predetermined time intervals ranging from 1 to 60 min, and the remaining method of concentration was analyzed accordingly. The effect of ionic strength on metformin removal was investigated by introducing different concentrations of NaCl (0–100 mM) into the solution. The metformin removal efficiency (%*R*) and adsorption capacity (*q_e_*, mg g^−1^) were calculated using Equations (1) and (2), respectively [53,54]:

(1) $$\%R\;=\;\frac{C_{o}-C_{e}}{C_{o}}\;\times \;100$$

(2) $$q_{e}\;=\;\frac{C_{o}-C_{e}}{m}\;\times \;V$$

The initial concentration of metformin is denoted as *C_o_* (mg L^−1^), while *C_e_* (mg L^−1^) represents the equilibrium concentration after treatment. The adsorbent mass is indicated by *m* (g), and *V* (L) refers to the volume of the solution.

## 3. Results and Discussion

### 3.1. Surface Morphology and Characterization

**Figure 2:**
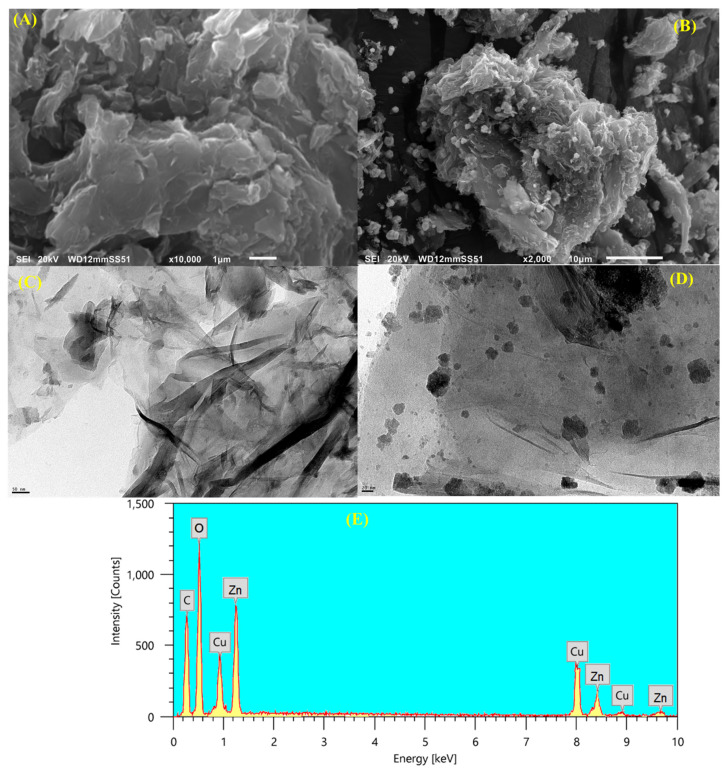
(**A**,**B**) SEM micrographs and (**C**,**D**) HRTEM images illustrating the morphologies of CGO and the Cu/ZnO@CGO nanocomposite, along with (**E**) the EDX spectrum of the Cu/ZnO@CGO nanocomposite.

The surface features of the CGO nanosheets and the Cu/ZnO@CGO nanocomposite were examined using high-resolution transmission electron microscopy (HRTEM) and scanning electron microscopy (SEM), with the corresponding images shown in Figure 2A–D. The SEM image of CGO (Figure 2A) reveals a vertically aligned, densely stacked sheet-like structure exhibiting characteristic wrinkles. In contrast, the SEM image of Cu/ZnO@CGO (Figure 2B) shows small, draped spherical features attributed to the embedded Cu and ZnO nanoparticles. The HRTEM image of CGO (Figure 2C) displays thin, wrinkled films, indicative of aggregated graphene oxide sheets, likely resulting from covalent bonding interactions. Meanwhile, Figure 2D presents the HRTEM image of Cu/ZnO@CGO, where darker spots are clearly visible on the CGO matrix, confirming the presence of Cu and ZnO nanoparticles. The 3D structure of the nanocomposite appears more porous, irregular, and rougher, suggesting effective integration of Cu and ZnO onto the CGO surface. Dynamic light scattering (DLS) measurements indicated that the Cu/ZnO@CGO nanocomposite exhibited an average particle diameter of 37.5 ± 4.1 nm. The Cu/ZnO@CGO nanocomposite exhibited a BET specific surface area of 587.33 m^2^ g^−1^, with a mean pore width of 24.721 nm and a total pore volume of 0.635 cm^3^ g^−1^. The volume of gas adsorbed at STP corresponding to monolayer formation on the surface was 23.6512 cm^3^ g^−1^.

EDX analysis was employed to confirm the elemental composition of the synthesized Cu/ZnO@CGO nanocomposite (Figure 2E). The spectrum reveals characteristic peaks corresponding to C (at 0.28 keV), O (at 0.52 keV), Zn (1.01 and 8.63 keV for Zn L and Kα transitions, respectively), and Cu (at 0.93, 8.04, and 8.90 keV for Cu L, Kα, and Kβ transitions, respectively). The presence of C and O originates from the CGO matrix and oxide functional groups, verifying the carbon-based support structure. The well-defined Zn and Cu peaks confirm the successful incorporation and coexistence of ZnO and Cu species within the nanocomposite without detectable impurity elements. Moreover, the clear separation and intensity of the metal peaks indicate a homogeneous distribution of Zn and Cu over the CGO surface, supporting the effective formation of the Cu/ZnO@CGO nanocomposite.

**Figure 3:**
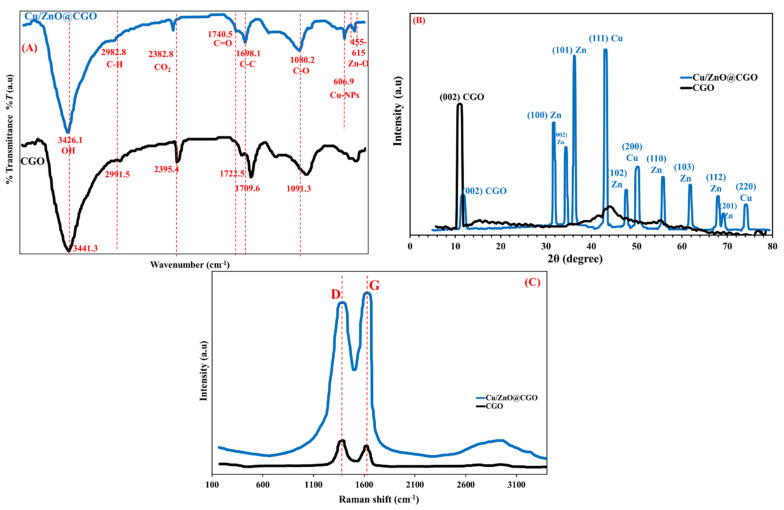
FTIR (**A**), XRD (**B**) and Raman spectra (**C**) of Cu/ZnO@CGO nanocomposite.

**Figure 4:**
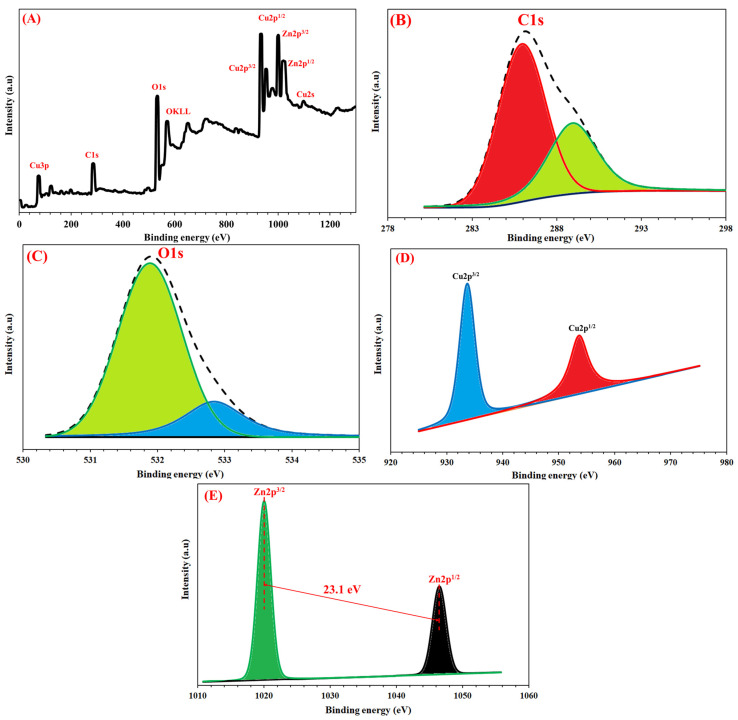
XPS analysis of the Cu/ZnO@CGO nanocomposite: survey spectrum (**A**), along with high-resolution spectra of C1s (**B**), O1s (**C**), Cu2p (**D**), and Zn2p (**E**).

The FT-IR spectra of CGO and Cu/ZnO@CGO are presented in Figure 3A. A peak at 1698.1–1709.6 cm^−1^ corresponds to C=C stretching in aromatic structures, while the broad absorption band at 3426.1–3441.3 cm^−1^ is attributed to O–H stretching vibrations [55]. The sharp band at 1722.5–1740.5 cm^−1^ is assigned to carboxyl groups in the CGO framework. A distinct absorption peak at 606.9 cm^−1^ is linked to the Cu nanoparticles, whereas characteristic Zn-O vibrations are observed around 455–615 cm^−1^. These findings confirm successful anchoring of Cu and ZnO nanoparticles to the CGO surface. XRD patterns (Figure 3B) further validated the structural composition of the Cu/ZnO@CGO composite. The pristine CGO displayed a (002) diffraction peak at 11.2°, while the nanocomposite exhibited additional peaks at 74.2°, 50.3°, and 43.3°, related to the (220), (200), and (111) planes of Cu nanoparticles, in agreement with standard Cu reference data [56]. A broader (002) peak at approximately 10.8° also appeared, associated with modified CGO sheets. Peaks corresponding to ZnO nanoparticles were observed at 31.8° (100), 34.4° (002), 36.4° (101), 47.9° (102), 56.0° (110), 61.9° (103), 68.1° (112), and 69.3° (201), aligning well with the ZnO standard diffraction data [57]. The absence of any extraneous peaks indicates the high purity of the synthesized nanocomposite. Raman spectroscopy was employed to examine structural defects and disorder within the CGO sheets. Both CGO and Cu/ZnO@CGO exhibited prominent D and G bands at 1396 cm^−1^ and 1614 cm^−1^, respectively (Figure 3C). The D band corresponds to the vibrational breathing modes of sp^3^-hybridized carbon atoms arising from structural imperfections, whereas the G band is attributed to the *E*_2_g phonon vibration of sp^2^-hybridized carbon frameworks. The elemental composition and chemical states present in the Cu/ZnO@CGO nanocomposite were further analyzed by X-ray photoelectron spectroscopy (XPS), as shown in Figure 4A. The C1s spectrum (Figure 4B) displayed peaks at 288.9 eV and 286.0 eV, corresponding to the carboxyl group (-COOH) and C=O/C–O–C and C–C/C=C bonds of the aromatic CGO rings, respectively [58]. In the O1s region (Figure 4C), two peaks were detected: one at 531.2 eV associated with lattice oxygen in ZnO, and another at 533.0 eV related to oxygen in surface groups such as C=O, O–C–O, and adsorbed hydroxyls. The Cu2p spectrum (Figure 4D) showed two significant peaks at 933.8 eV (Cu2p^3/2^) and 953.7 eV (Cu2p^1/2^), separated by a 20-eV spin–orbit split, characteristic of Cu species [59]. The Zn 2p spectrum (Figure 4E) revealed peaks at 1045.0 eV and 1022.0 eV, corresponding to Zn 2p^1/2^ and Zn 2p^3/2^, respectively, consistent with Zn^2+^ in ZnO and exhibiting a spin–orbit separation of 23.1 eV.

### 3.2. Impact of pH

**Figure 5:**
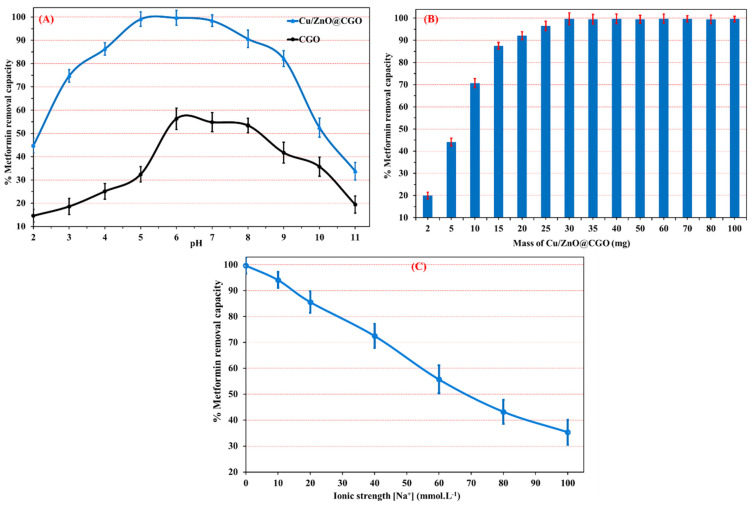
(**A**) Influence of the initial solution pH, (**B**) Cu/ZnO@CGO dosage, and (**C**) solution ionic strength on metformin removal efficiency under optimized experimental conditions (initial metformin concentration = 20 mg L^−1^; temperature = 298.15 K; nanocomposite dose = 30 mg; data expressed as mean ± standard deviation, n = 3).

The adsorption performance of metformin onto the Cu/ZnO@CGO nanocomposite is strongly influenced by solution pH, as it governs the interactions between the functional groups of metformin and the active sites on the surface of the ternary nanocomposite. The effect of pH on adsorption behavior is illustrated in Figure 5A. As the pH increases from 2 to 11, the sorption capacity of Cu/ZnO@CGO rises markedly, reaching a maximum at pH 6.0, followed by a pronounced decrease under alkaline conditions.

Metformin is a biguanide derivative containing two methyl groups at the N-1 position. Its molecular structure comprises primary, secondary, and tertiary amine groups, in addition to two imine functionalities (C=N–H). Metformin exhibits two dissociation constants (p*K*_a1_ = 2.8 and p*K*_a2_ = 11.5), indicating that it predominantly exists as a hydrophilic cation under physiological conditions [60]; Figure 1. Based on its chemical structure and p*K*_a_ values, metformin carries an overall positive charge at acidic pH, adopts a zwitterionic form at neutral to mildly acidic pH, and becomes negatively charged under basic conditions.

At acidic pH levels (pH < 4.0), adsorption is mainly governed by hydrogen bonding, π–π stacking, and cation–π interactions. Electron donor–acceptor (EDA) interactions arise through π–π stacking between metformin molecules and the conjugated π-electron system of CGO, involving overlap with the aromatic domains of the carbon framework. In addition, cation–π interactions occur between the protonated nitrogen-containing groups of metformin (primary, secondary, and tertiary amines, as well as imine groups) and the π-electrons of the aromatic rings within CGO. The polar surface of ZnO nanoparticles further facilitates adsorption via hydrogen bonding and electrostatic attractions.

The enhanced adsorption observed at neutral to moderately acidic pH values (pH 4.0–9.0) can be primarily attributed to surface complexation reactions involving Cu and ZnO nanoparticles with the imine groups and primary and tertiary amines of metformin through ligand-exchange mechanisms. These interactions are particularly favored under conditions where surface hydroxyl groups on ZnO nanoparticles undergo deprotonation.

At strongly alkaline pH values (pH > 10), the observed decline in adsorption capacity is mainly associated with the weakening of cation–π interactions, hydrogen bonding, and π–π stacking. This reduction is largely due to the diminished surface complexation between Cu nanoparticles and nitrogen-containing functional groups of metformin, resulting from deprotonation of ammonium species under basic conditions. The above findings were further corroborated by zeta potential analysis, which demonstrated that the surface charge of the Cu/ZnO@carboxylated graphene oxide (CGO) nanocomposite is strongly pH-dependent and plays a critical role in governing electrostatic interactions with metformin molecules. At pH 6.0 (near-neutral conditions), progressive deprotonation of surface carboxyl and phenolic groups on the nanocomposite results in the development of a highly negative surface charge, reaching an optimal zeta potential of −50.8 mV, at which the adsorption capacity is maximized. Under these conditions, metformin containing protonated amine and imine functionalities due to its acid dissociation constants (pKₐ = 2.8 and 11.5), exists predominantly as a hydrophilic monocation, enabling strong electrostatic attraction to the negatively charged Cu/ZnO@CGO surface and thereby enhancing its removal efficiency.

### 3.3. Impact of Mass and Ionic Strength

The effect of adsorbent dosage on metformin uptake by the Cu/ZnO@CGO nanocomposite was investigated by varying the nanocomposite mass from 2 to 100 mg at pH 6.0, using a contact time of 25 min and an initial metformin concentration of 20 mg.L^−1^ (Figure 5B). The results demonstrate that increasing the adsorbent dose from 5 to 20 mg led to a substantial enhancement in metformin removal efficiency, rising from 45% to 95%. This improvement is attributed to the increased availability of active adsorption sites on Cu/ZnO@CGO, which promotes greater interaction with metformin molecules in solution [61]. Beyond a dosage of 25 mg, the removal efficiency reached a plateau, remaining nearly constant at 99.7% ± 3.1. Consequently, a nanocomposite mass of 25.0 mg was selected as the optimal dosage for subsequent experiments.

The impact of ionic strength on adsorption performance was evaluated by introducing different concentrations of NaCl into the metformin–Cu/ZnO@CGO suspension, as illustrated in Figure 5C. A gradual decrease in metformin removal efficiency was observed with increasing NaCl concentrations in the range of 10–100 mM at an initial metformin concentration of 20.0 mg·L^−1^. Notably, at an NaCl concentration of 50 mM, the adsorption efficiency declined by approximately 50–53%, indicating that elevated ionic strength adversely affects metformin uptake. Electrostatic screening and cation–π bonding phenomena are used to explain this behavior. The solution’s increased ionic strength would prevent the electrostatic interactions necessary for metformin’s surface complexation with copper nanoparticles. Additionally, the surface charge sites are electronically screened upon the addition of Na^+^ ions, which reduces the cation–π bonding between the aromatic ring of CGO and the positively charged protonated amine moiety present in metformin [62,63,64,65].

### 3.4. Influence of Cu- and ZnO-Supported Nanoparticles on the Adsorption Performance of the Cu/ZnO@CGO Toward Metformin

**Figure 6:**
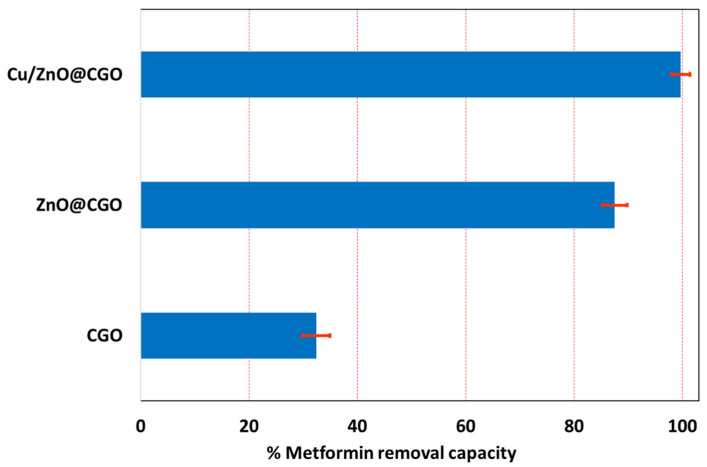
Impact of Cu and ZnO nanoparticles on the removal capacity for metformin-Cu/ZnO@CGO adsorption system.

Sorption experiments were carried out for CGO, ZnO@CGO and Cu/ZnO@CGO nanocomposites at pH = 6.0 to assess the Cu and ZnO nanoparticles’ adsorption performance in the innovative nanocomposite. Figure 6 presents the results of this comparative study. The incorporation of supported Cu/ZnO nanoparticles led to a substantial enhancement in metformin removal efficiency, with an increase of 67.3% observed at pH 6.0. This pronounced improvement in adsorption performance can be primarily attributed to the presence of Cu and ZnO nanoparticles and may be explained by two main factors. First, the supported nanoparticles inhibit aggregation of the CGO component, thereby markedly increasing the available surface area of the Cu/ZnO@CGO nanocomposite. Second, within the pH range of 5–8, the formation of Cu–metformin and Zn–metformin surface complexes on the nanocomposite further contributes to the enhanced adsorption capacity. Also, the hydrogen bonding between the -NH and -NH_2_ groups of metformin and the surface hydroxyls of ZnO NPs, as well as the abundant oxygen functional groups in CGO, contribute to a great extent to the metformin removal capacity. These findings clearly indicate that Cu/ZnO nanoparticles are a critical contributor to the metformin removal efficiency of the synthesized Cu/ZnO@CGO nanocomposite.

### 3.5. Sorption Mechanisms

**Figure 7:**
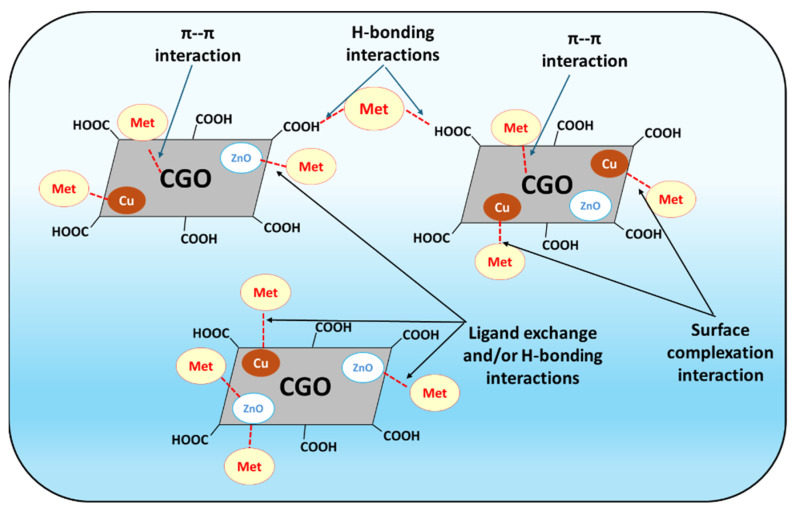
Schematic representation of the different interaction pathways between metformin molecules and the Cu/ZnO@CGO nanocomposite.

Metformin uptake onto the Cu/ZnO@CGO is governed by a synergistic interplay of multiple physicochemical interactions. Numerous functional groups, including carboxyl (-COOH), hydroxyl (-OH), and epoxy (-C-O-C), are present in CGO, which increases surface reactivity and offers many binding sites. ZnO-NPs have polar surfaces that facilitate contact through hydrogen bonding and electrostatic attraction. And the Cu-NPs allow for metal–ligand coordination or π-complexation. Five distinct interaction mechanisms governing the sorption of metformin onto Cu/ZnO@CGO, depending on the solution pH, have been identified, as illustrated in Figure 7 [66,67]. First, Cu nanoparticles coordinate with the two available imine (–C=NH) groups of metformin to generate stable five-membered chelate structures, and they also interact with the free amino group to form Cu–metformin surface complexes. Furthermore, the presence of Cu nanoparticles inhibits aggregation of the CGO component, leading to an increase in the effective surface area of the Cu/ZnO@CGO nanocomposite and, consequently, an enhancement in metformin adsorption capacity. The results discussed in Section 3.4 confirm that surface complexation involving Cu species represents the dominant interaction mechanism governing metformin adsorption onto Cu/ZnO@CGO [68]. The second interaction is the ligand exchange through ZnO surfaces that can bind metformin, especially in the pH range of 5–7 where surface hydroxyl groups can deprotonate. The hydrogen bonding between the -NH and -NH_2_ groups of metformin and the surface hydroxyls of ZnO NPs as well as the abundant oxygen functional groups in CGO (-COOH, -OH, and -C–O–C) is the third mechanism. The fourth one is the interaction between the aromatic rings in CGO and the π–π electron donor–acceptor (EDA). Cation–π bonding is the fifth mechanism, which is mainly driven by the electrostatic interactions between the enduring quadrupole of the plenty of π-electrons in the aromatic matrix in the CGO and the protonated nitrogen groups of metformin [67,69,70]. Incorporation of Cu/ZnO metallic nanoparticles into the Cu/ZnO@CGO structure provides a significant advantage in promoting metformin adsorption efficiency.

### 3.6. Sorption Kinetics

**Figure 8:**
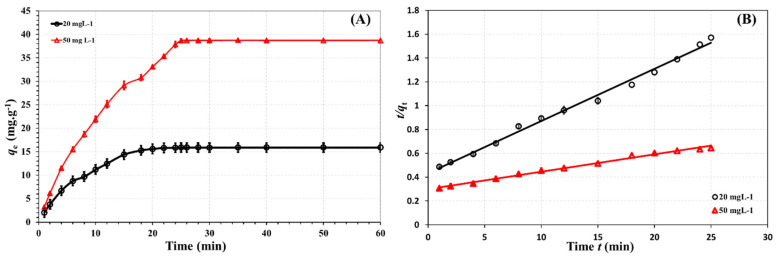
Impact of contact time (**A**), PSO kinetic model (**B**) for the metformin adsorption removal by Cu/ZnO@CGO for metformin at two different concentrations (20 and 50 mg. L^−1^) at 298.15 K.

Two different initial concentrations (50 and 20 mg. L^−1^) were used in this study to investigate the effect of contact duration on the adsorptive removal of metformin by the Cu/ZnO@CGO. The metformin adsorption capacities at the investigated concentrations were displayed versus contact time in Figure 8A. Equilibrium between the tertiary nanocomposite and metformin was achieved within 25 min, and the adsorption process was found to occur via two successive kinetic stages. The initial adsorption phase proceeded rapidly and was essentially completed within the first 10 min, followed by a second, slower stage that required an additional 10 min to reach completion. This two-step adsorption behavior was consistently observed across all three investigated initial metformin concentrations. The PSO or pseudo-second-order kinetic model was applied to evaluate how well the experimental results fit the kinetic behavior of the Cu/ZnO@CGO–metformin adsorption system. According to the model, chemisorption is the rate-controlling step for the removal of several pharmaceutical contaminants from water [71,72]. The PSO equation’s linear version is as follows:

(3) $$\frac{t}{q_{t}}\;=\;\frac{1}{k_{2}\;q_{e}^{2}}\;+\;\frac{1}{q_{e}}\;t$$

Here in, *q_e_* and *q_t_* (mg g^−1^) are the number of mg of metformin adsorbed at equilibrium and time *t* per mg of Cu/ZnO@CGO nanosorbent, respectively, and *k* (g mg^−1^ min^−1^) is the second-order rate constant. The PSO rate expression is applicable, and there is a significant correlation between the parameters, as shown by the high correlation coefficients (*R*^2^ > 0.99) of the linear graph of *t*/*q_t_* vs. *t* (Figure 8B) for all the investigated concentrations. Furthermore, the adsorption capacity values derived from the experimental data were found to be in close agreement with the calculated values. (22.9 and 69.3 mg g^−1^), and the PSO model (22.83 and 68.97 mg g^−1^, respectively).

The linearized models were employed primarily for comparative assessment, not as the sole basis for mechanistic or quantitative conclusions. The excellent agreement between experimental data and model predictions, reflected by very high determination coefficients (*R*^2^ > 0.99 for PSO and 0.9933 for Langmuir), indicates that potential bias introduced by linearization does not materially impact the interpretation of adsorption behavior in this system. Furthermore, our principal findings, including equilibrium time, adsorption capacity, and mechanistic dominance of chemisorption pathways, were drawn from the experimental data trends rather than solely from mathematical fitting outputs, ensuring the reliability of our conclusions.

### 3.7. Cu/ZnO@CGO Reusability

**Figure 9:**
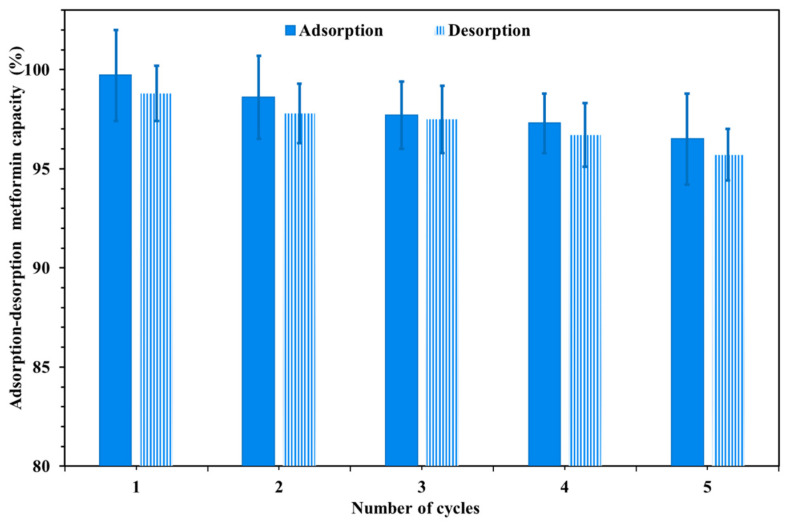
Recycling performance of the metformin–Cu/ZnO@CGO adsorption system.

Any adsorbent must have excellent reusability and a reasonable adsorption capacity for practical applications. The findings of this study (Figure 9) showed that nearly all the metformin anchored to the Cu/ZnO@CGO nanocomposite’s surface could be desorbed by 0.15 *M-*HCl solution, which maintained consistency for up to five adsorption–desorption cycles. The adsorption capacity began to decline after five cycles. After extracting the recycled Cu/ZnO@CGO nanocomposite, we weighed it. Following the five cycles of Cu/ZnO@CGO nanocomposite regeneration, we did not observe any significant mass loss. Furthermore, the percentage extraction values of metformin from the recycled Cu/ZnO@CGO were comparable to those of the original nanocomposite. The sorption capacity of the Cu/ZnO@CGO nanocomposite remains high for up to six cycles with only minor loss of effectiveness, as demonstrated by the efficacy of the metformin removal from the sixth and first cycles of desorption, which ranged from 99.7% to 95.7% (Figure 9). Cu/ZnO@CGO’s outstanding desorption efficiency and reusability indicated that the nanocomposite might be used effectively as an affordable and active nanosorbent to extract metformin from aqueous environments.

### 3.8. Sorption Isotherms

The adsorption isotherm of metformin onto the Cu/ZnO@CGO nanosorbent provides valuable insight into the adsorption mechanism, surface characteristics, and the affinity between the adsorbate and adsorbent. To interpret the adsorption behavior, the experimental data were analyzed using Langmuir, Freundlich, and Temkin isotherm models. The corresponding linearized forms of these models are expressed as follows:

Langmuir:

(4) $$\frac{1}{q_{e}}\;=\;\frac{1}{q_{m}}\;+\;\frac{1}{K_{L}\;q_{m}\;C_{e}}$$

Freundlich:

(5) $$\ln\;q_{e}\;=\;\ln K_{F}\;+\;\frac{1}{n}\;\ln\;C_{e}$$

Temkin:

(6) $$\;q_{e}\;=\;K_{T}\ln C_{e}\;+\;K_{T}\;\ln\;f$$

In these models, *C_e_* represents the equilibrium concentration of metformin in solution (mg L^−1^), while *q_m_* (mg g^−1^) denotes the maximum adsorption capacity of the nanocomposite. The parameter n corresponds to the Freundlich linearity constant, which indicates the favorability of the adsorption process, whereas *K_L_*, *K_F_*, and *K_T_* are the adsorption constants associated with the Langmuir, Freundlich, and Temkin models, respectively.

The Langmuir isotherm is commonly used to describe a reversible adsorption process involving monolayer coverage on a homogeneous adsorbent surface. In contrast, the empirical Freundlich model accounts for adsorption occurring on heterogeneous surfaces with the possible formation of multilayer structures. According to the Temkin model, the adsorption mechanism is governed by chemisorption, where strong electrostatic interactions arise between oppositely charged species [72].

**Figure 10:**
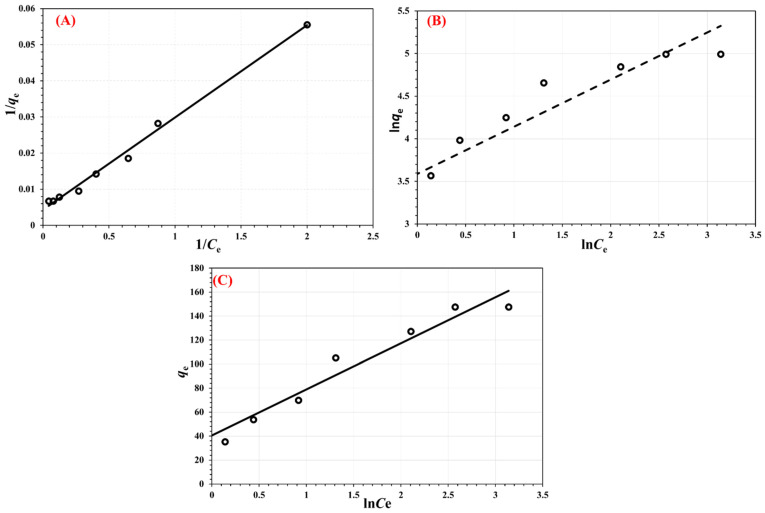
Langmuir (**A**), Freundlich (**B**), and Temkin (**C**) adsorption isotherm models describing metformin adsorption onto the Cu/ZnO@CGO nanocomposite at 298.15 K.

The adsorption isotherms for metformin removal using the Cu/ZnO@CGO nanocomposite, fitted with the three isotherm models, are presented in Figure 10A–C, while the corresponding fitting parameters are summarized in Table 1. The Freundlich (*R*^2^ = 0.9021) and Temkin (*R*^2^ = 0.936) models showed good agreement with the experimental data; however, the Langmuir model exhibited the highest correlation coefficient (*R*^2^ = 0.9933), indicating superior fitting performance. Based on the Langmuir model, the theoretical maximum adsorption capacity (*q_m_*) for metformin was estimated to be 232.56 mg g^−1^.

At pH 6.0, the increased proportion of deprotonated amine and imine functional groups in the metformin molecule enhances surface complexation with Cu nanoparticles, thereby improving adsorption efficiency. In addition to surface complexation, hydrogen bonding, electrostatic interactions, and π–π electron donor–acceptor (EDA) interactions contribute to the overall adsorption mechanism. A comparison of the maximum adsorption capacities of the Cu/ZnO@CGO nanocomposite with those of previously reported adsorbents is provided in Table 1. The superior performance of the developed nanocomposite can be attributed to the functionalization of CGO with Cu nanoparticles, which facilitates the formation of inner-sphere surface complexes and significantly enhances the *q_m_* values.

### 3.9. Analytical Performance of Cu/ZnO@CGO Nanocomposite

Sorption experiments targeting the removal of metformin were conducted using tap water, river water, and commercially bottled drinking water to evaluate the adsorption performance of Cu/ZnO@CGO toward metformin. The corresponding results are summarized in Table 2. Notably, metformin removal efficiencies obtained in river water samples (98.8–99.9%) were markedly higher than those observed in spiked tap water and bottled drinking water. This enhanced removal behavior is likely attributed to the presence of humic substances in river water as well as variations in solution pH, which can influence sorption interactions [73,74].

## 4. Conclusions

For the first time, the development of a Cu-ZnO@carboxylated graphene oxide nanocomposite was achieved and utilized to rapidly and efficiently remove the medication metformin from environmental water samples. Two benefits of the incorporated Cu-NPs add to the nanocomposite’s characteristics. The first one has to do with the nanocomposite’s exceptional dispersibility and preservation of its two-dimensional structure, which are predicated on the Cu nanoparticles’ capacity to stop CGO from clumping together owing to the influence of π–π stacking. A further advantage pertains to the rapid and efficient metformin removal enabled by surface Cu–metformin complex formation. The adsorption of metformin onto Cu/ZnO@CGO was attributed to four different interactions controlled by the solution pH. In addition to intermolecular hydrogen bonding via the amine groups of metformin and ZnO, as well as the oxygen-containing functional groups in CGO, metformin interacts with CGO via π–π electron donor–acceptor (EDA) associations and cation–π interactions. The primary mechanism by which metformin interacts with Cu and ZnO nanoparticles in Cu/ZnO@CGO is surface complexation. According to the PSO kinetic model, the adsorption process occurs rapidly, and the metformin removal efficiency across various water samples from different aquatic environments was quite satisfactory (99.7 ± 3.4%). The newly developed nanocomposite exhibits remarkable metformin extraction capability, high enrichment factor, high adsorption rates, good precision, and excellent reusability.

## Figures and Tables

**Table 1:** The calculated fitting parameters of the Langmuir, Freundlich, and Temkin adsorption isotherm models for metformin uptake by the Cu/ZnO@CGO nanocomposite.

Langmuir			Freundlich			Temkin		
*q_m_* (mg g^−1^)	*K_L_* (L mg^−1^)	*R* ^2^	*K_F_* (L mg^−1^)	*n*	*R* ^2^	*K_r_*	*f*	*R* ^2^
232.56	0.1686	0.9933	36.11	1.8086	0.9021	38.385	2.870	0.9632

**Table 2:** Adsorptive removal of metformin from environmental water matrices using the Cu/ZnO@CGO nanocomposite.

Water Sample	Water Parameters		Spiked Conc. (mg L^−1^)	Detected Conc. (mg L^−1^)	Recovery (%)
	pH	Conductivity (μs cm^−1^)			
River water	7.83	324.0	5.2	5.14	98.8 ± 3.5
			10.4	10.27	99.8 ± 4.5
			20.0	19.88	99.4 ± 4.1
Tap water	7.13	81.3	5.0	4.98	99.6 ± 3.4
			10.0	9.97	99.7 ± 3.1
			20.0	19.95	99.8 ± 3.7
Bottled water	8.62	24.15	10.0	9.99	99.9 ± 1.5

## Data Availability

The original contributions presented in this study are included in the article. Further inquiries can be directed at the corresponding author.
